# The Next Generation of Medical Decision Support: A Roadmap Toward Transparent Expert Companions

**DOI:** 10.3389/frai.2020.507973

**Published:** 2020-09-24

**Authors:** Sebastian Bruckert, Bettina Finzel, Ute Schmid

**Affiliations:** Cognitive Systems, University of Bamberg, Bamberg, Germany

**Keywords:** explainable artificial intelligence, interactive ML, interpretability, trust, medical diagnosis, medical decision support, companion

## Abstract

Increasing quality and performance of artificial intelligence (AI) in general and machine learning (ML) in particular is followed by a wider use of these approaches in everyday life. As part of this development, ML classifiers have also gained more importance for diagnosing diseases within biomedical engineering and medical sciences. However, many of those ubiquitous high-performing ML algorithms reveal a black-box-nature, leading to opaque and incomprehensible systems that complicate human interpretations of single predictions or the whole prediction process. This puts up a serious challenge on human decision makers to develop trust, which is much needed in life-changing decision tasks. This paper is designed to answer the question how expert companion systems for decision support can be designed to be interpretable and therefore transparent and comprehensible for humans. On the other hand, an approach for interactive ML as well as human-in-the-loop-learning is demonstrated in order to integrate human expert knowledge into ML models so that humans and machines act as companions within a critical decision task. We especially address the problem of *Semantic Alignment* between ML classifiers and its human users as a prerequisite for semantically relevant and useful explanations as well as interactions. Our roadmap paper presents and discusses an interdisciplinary yet integrated Comprehensible Artificial Intelligence (cAI)-transition-framework with regard to the task of medical diagnosis. We explain and integrate relevant concepts and research areas to provide the reader with a *hands-on-cookbook* for achieving the transition from opaque black-box models to interactive, transparent, comprehensible and trustworthy systems. To make our approach tangible, we present suitable state of the art methods with regard to the medical domain and include a realization concept of our framework. The emphasis is on the concept of Mutual Explanations (ME) that we introduce as a dialog-based, incremental process in order to provide human ML users with trust, but also with stronger participation within the learning process.

## 1. Introduction

**Figure 1:**
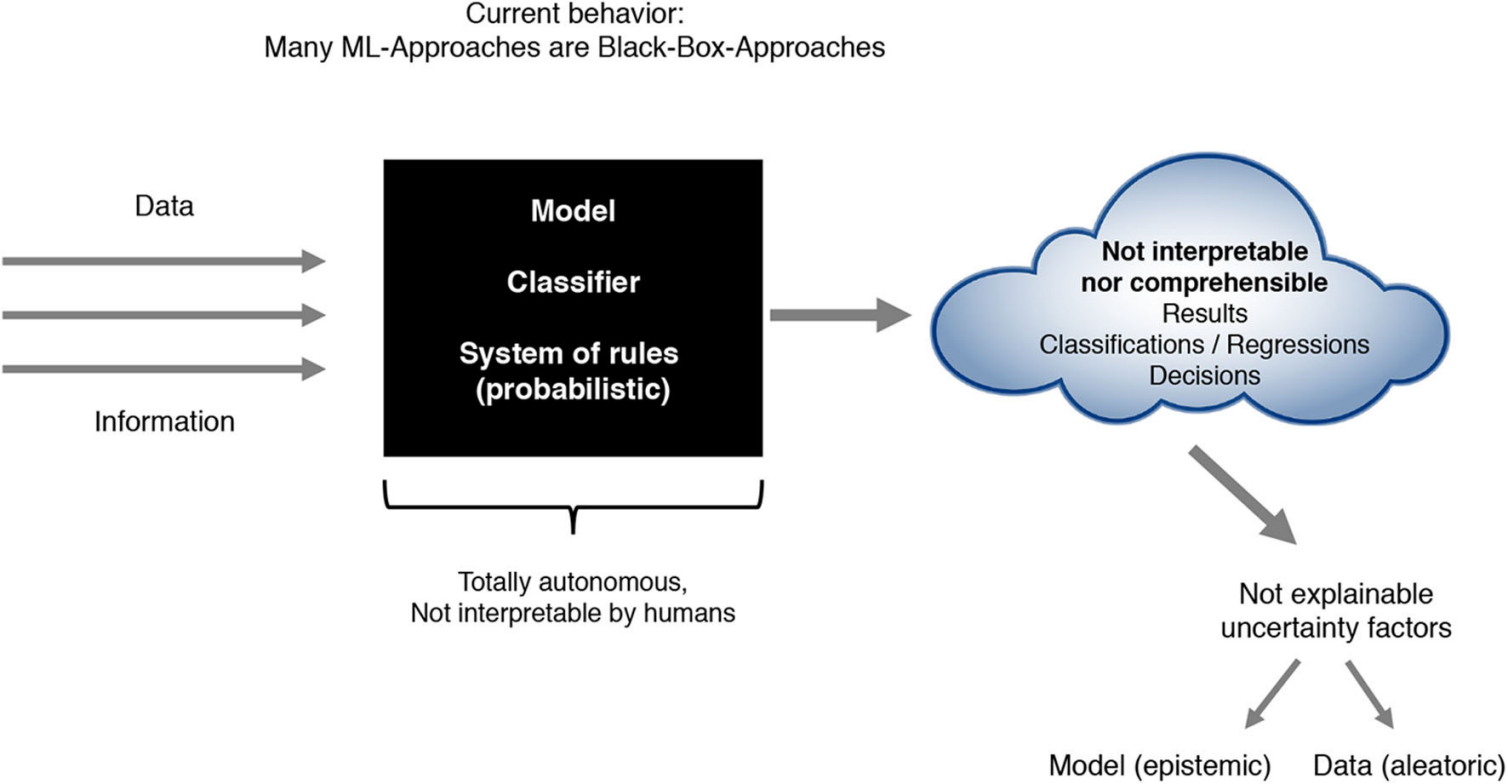
Present ML-approaches as black-box approaches.

Although modern ML approaches improved tremendously in terms of quality (prediction accuracy) and are able to even exceed human performance in many cases, they currently lack the ability to provide an explicit declarative knowledge representation and therefore hide the underlying explanatory structure (Holzinger et al., 2017). Due to this inability, modern ML approaches often result in black-box approaches—models and techniques, whose internal approach stays unknown and that just connect observable input- and output information without allowing an understanding nor an explanation of the way results have been produced (see Figure 1). Exactly that missing transparency makes it difficult for users of ML techniques to develop an understanding of the recommendations and decisions, which mostly constitutes an inherent risk (Sliwinski et al., 2017).

In a legal sense the question of legal security and liability security arises. Since the European General Data Protection Regulation (GDPR and ISO/IEC 27001) has entered into force in May, 2018, the relationship between AI and applicable law contains tremendous potential for clarification (Holzinger et al., 2017). As an example, the question of liability arises, especially if third parties suffer damages that are caused by recommendations or decisions made by ML approaches. According to latest jurisprudence, software architects, software developers as well as users are only liable for their actions and artifacts if a certain behavior of the system would have been predictable (Burri, 2016).

**Figure 2:**
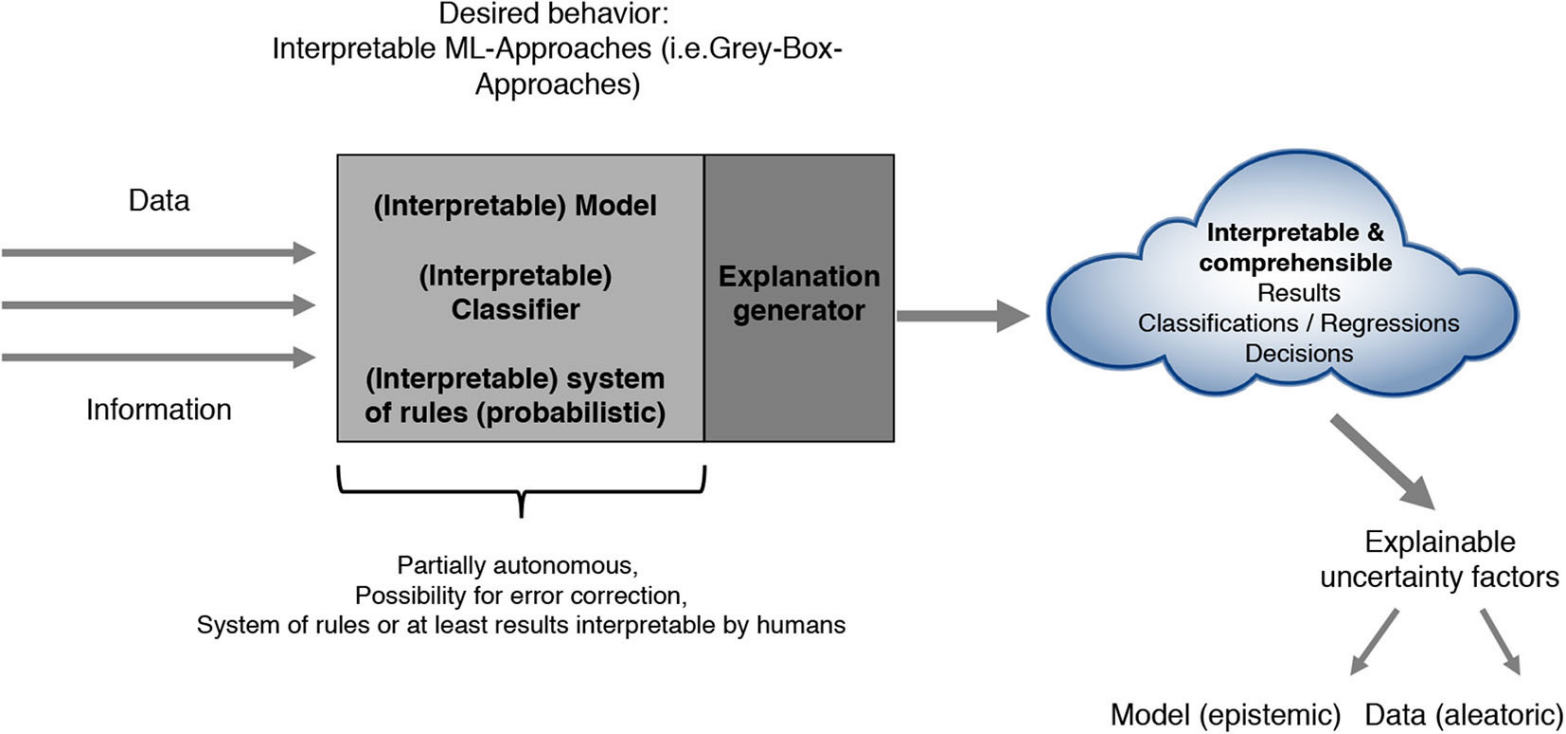
Future comprehensible ML-approaches (i.e., gray-box approaches).

Most current architectures as described in Figure 1 often lead to several problems. On the one hand, the system of internal rules itself often is not interpretable by humans. On the other hand, the ML results in terms of classification, regression or policy outputs are not comprehensible nor explainable due to biases and uncertainty introduced by the used model, the data or other factors. In addition, human experts have difficulties in integrating their expert knowledge into the learning process. All of the just mentioned points of criticism have led to a steadily increasing importance of the research areas Explainable Artificial Intelligence (xAI), Interpretable Machine Learning (iML) and Interactive ML that we summarize and refer to as Comprehensible Artificial Intelligence (cAI). These primarily aim at developing approaches that in addition to a precise prediction accuracy fulfill concepts like interpretability, explainability, confidence including stability and robustness, causality, interactivity, liability and liability security in a legal sense, socio-technical and domain aspects, bias awareness as well as uncertainty handling. The intention of cAI can be characterized by either achieving interpretability regarding the models or by making at least the results itself understandable and explainable and therefore interpretable (see Figure 2). We develop and present our cAI framework with regard to the application of ML for medical diagnosis. Since medical diagnosis comprises a complex process relevant for many succeeding medical sub-disciplines with high human involvement, diagnostic decisions not only need to be done accurately and precisely, but also in a comprehensible and trustworthy manner. Convolutional neural networks can be used to demonstrate the current trade-off between ML performance and interpretability. Such deep learning approaches often used for image-based medical diagnosis perform well in terms of prediction accuracy, but the models as well as their decisions cannot be interpreted easily without further investigations.

## 2. Method/Design

**Figure 3:**
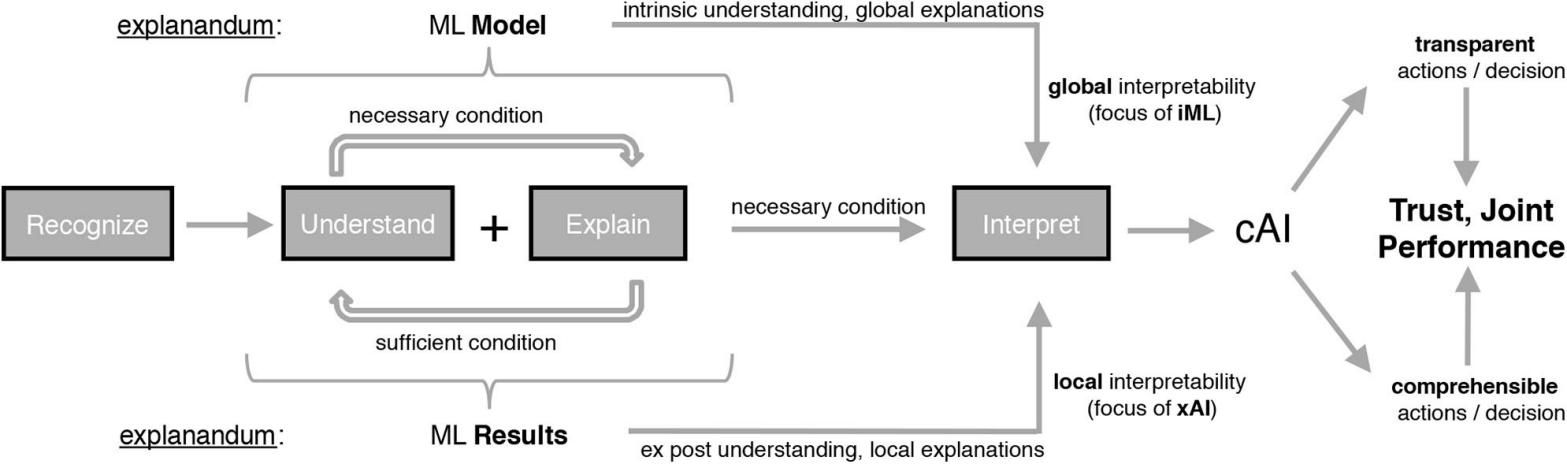
Derivation of cAI from iML and xAI considering the underlying cognitive concepts.

In order to address the shortcomings mentioned above, we first provide an overview of the cognitive concepts that are used in the course of this paper to differentiate between different research branches (see Figure 3). The cognitive concept of interpretation can be seen as the key concept, whose different shapings can be used as a criterion for differentiation of iML and xAI. From a philosophical and hermeneutical perspective, understanding and explaining are correlated terms and sometimes considered as symmetric cognitive concepts (Schurz, 2002). Having recognized and understood an issue therefore leads to having an explanation for it, and, reaching the state of understanding comes with having generated explanations. Thus the concept of understanding can be seen as necessary and sufficient condition for explaining and explaining represents a sufficient condition for understanding. Both concepts, understanding and explaining, in combination constitute a necessary condition for interpretation. iML and xAI differ in the explanandum as well as in the nature of desired interpretability, which the authors from Adadi and Berrada (2018) call the *scoop of interpretability*.

The task of making classifications, regressions or derived policies of an ML approach interpretable, contains sub-tasks like understanding and explaining as described in Figure 3. *Understanding*, which means recognizing correlations (context) in an intellectual way, can be seen as the bridge between human recognition and decision and is therefore the basis of explanation. Humans are performing really good in understanding a context and based on this generalizing from observations, whereas there is a long way still to go for AI especially in terms of contextualizing. On the basis of understanding a context, the explanation task, in addition, includes making the reasons of observed facts by stating logical and causal correlations comprehensible for humans (Holzinger, 2018). We draw a distinction between the attributes explainable and explicable within the AI context in stating that making facts explicable is a sub-task of the explanation task, meaning that purely explicating facts is not enough for humans to build an understanding. In terms of our cAI terminology (see Figure 3), ML models and results need to be explicable so that they are transparent to human users, but they need to be explainable for being comprehensible, too. We therefore refer to explicability as a property, which forms the basis for explainability and states that something potentially can(!) be explained, but it doesn't necessarily correspond to the concrete explanation for a certain set of facts in rationale terms. The focus of explaining can be differentiated regarding the explanation of the reasoning, the model or the evidence for the result (Biran and Cotton, 2017). However, in all cases, the goal of the explanation task can be seen as updating the humans' mental models (Chakraborti et al., 2018), where good explanations must be relevant to a, potentially implicit, human question as well as relevant to the mental model of the explainee (Miller, 2019).

Explanations can provide a valuable basis for providing transparency and comprehensibility regarding systems' decisions and therefore can lead to increased trust of ML users (Pu et al., 2011; Prahl and Swol, 2017; Miller, 2019). A high level of initial trust in ML systems, which often decreases rapidly in case of erroneous or unexpected reactions (Madhavan and Wiegmann, 2007), as well as interaction and influencing possibilities might be an acceptance criterion for the usage of such systems (Schaefer et al., 2016). As illustrated in Figure 3, we distinguish between two different shapings of the cognitive concept of interpretation—namely iML and xAI, which differ in the kind of understanding as well as in the way explanations are revealed. In our opinion, iML focuses on using or generating global interpretability by providing intrinsic—*ex ante*—understanding of the whole logic of the corresponding models (Adadi and Berrada, 2018). Global explanations therefore relate to the inner functioning of models, meaning the entire and general behavior in terms of the entire reasoning describing HOW the systems work internally. Hence, the scoop of this type of interpretability is to inform about the global effects giving some indication on the real concepts that a system has learned. The explanandum is therefore the ML model itself where we consider the *rules of reasoning* as the explanans giving information about how all of the different possible outcomes are connected to the inputs. On the other hand, we see xAI's focus more on enabling local interpretability by providing an *ex post*-understanding of the model's specific behavior. Local explanations for individual decisions or single predictions strive for making the input-output-correlations clear to the user without the need for knowing the model's internal structure (Adadi and Berrada, 2018). Thus, the scoop of this type of interpretability is to make justifications WHY a model produced its output in the way it did. The explanandum is therefore an individual ML result or a group of results where we see the occurrences, importances, and correlations of input features as the explanans giving information about the logical and causal correlations of inputs and outputs. The two dimensions spanned by cAI, in our understanding of interpretability, namely transparency and comprehensibility (see Figure 3), might aim at different requirements of different kind of users. Therefore, we refer to transparency as a property especially relevant to domain or ML experts that are not solely interested in why a certain output was made but also trying to explore the nature and characteristics of the underlying concepts and its context. In contrast, we refer to comprehensibility as a requirement raised particularly by humans that are directly affected by the outputs and the correlated consequences trying to understand why a specific decision was made. We define the overall objective of cAI as developing transparent and comprehensible AI systems that humans can trust in as well as improving the systems' “joint performance,” both by means of global interpretability (iML) in combination with local interpretability (xAI). Depending on the domain and the ML problem to be solved an adaptive combination of white-box approaches and black-box approaches with connected explanation generators and interfaces (gray-box approach) will be necessary in order to reach cAI.

**Figure 4:**
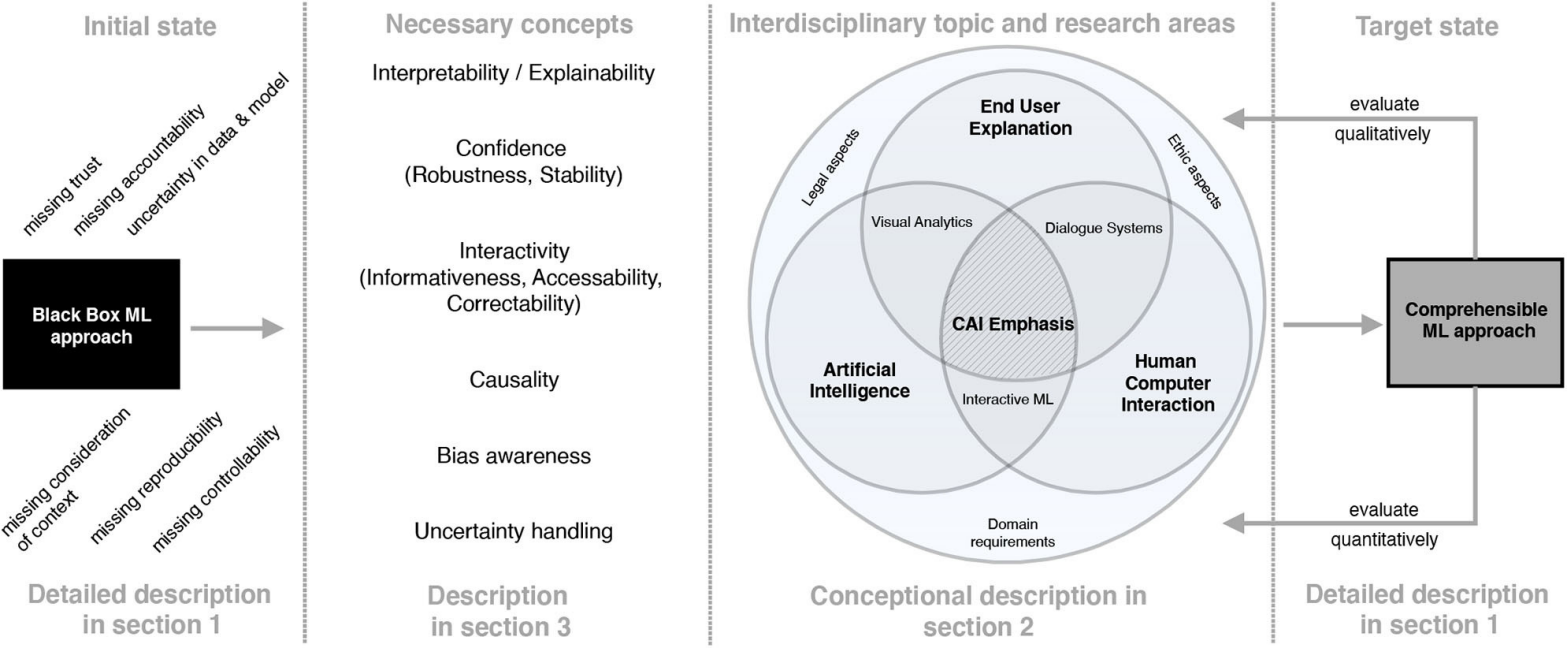
cAI transition framework using interdisciplinary concepts, approaches and measures to reach next level of AI.

Figure 4 illustrates our suggestion for a possible transition framework, which includes interdisciplinary concepts, approaches and measures to reach cAI and thus the next level of transparent and interactive companions for decision support. As discussed, current ML approaches lack conceptual properties like interpretability of the model as well as the results. Additionally, missing reproducibility of ML predictions and the according explanations imposes requirements on a concept called *confidence*, which the authors from Arrieta et al. (2020) refer to as a generalization of robustness and stability of ML approaches. Furthermore and due to missing interpretability, state of the art ML systems often do not provide any possibility for human interaction, since humans are not able to understand the rules the system has learned. Therefore, any correction of erroneous rules or any inclusion of domain-specific knowledge through human experts (i.e., physicians) is not possible. In addition, the points of criticism mentioned so far also lead to tremendous potential for clarification in terms of the relationship between AI and applicable law. Legal security and liability security will play a crucial role in the near future. As an example, in the medical domain the question of liability arises, especially if a patient suffers damages that are caused by a medical treatment of a physician who acted on the recommendation of an ML approach. Additionally, we consider socio-technical and domain aspects as other important conceptual properties, since in most cases ML pipelines need to be adapted to the according context of the problem to be solved. In the same way, explanation and interpretation techniques need to be in accordance with the individual domain and social as well as ethical requirements. Causality is another necessary concept (Pearl, 2009) and refers to making underlying mechanisms transparent beyond computing correlations (Holzinger et al., 2019) to derive the *true* reasons that lead to a particular outcome. Therefore, causality depends on available interpretability and explainability of models. This requirement as precondition to causality can be referred to as causability and is currently examined in the context of explanation evaluation, especially for the medical domain (Holzinger et al., 2019). Analogously to our differentiation between explicability and explainability, we strongly agree with the authors from Holzinger et al. (2019) that results gained from explainable and interpretable models should not only be usable but also useful to humans. In this regard they refer to Karl Popper's hypothetical deductive model in order to derive facts from laws and conditions in a deductive manner by causal explanations. Bias awareness as further concept focuses on avoiding ML-related biases in predictive modeling like sample bias, exclusion bias, label bias, bias in ground truth as well as other more general biases like observer bias, prejudice bias and measurement bias. A remedy can be to use techniques such as FairML, which is a toolbox for diagnosing bias in predictive modeling (Sgaard et al., 2014; Adebayo, 2016). Uncertainty is another concept that should be taken into account. In ML two types of uncertainty are distinguished (Kendall and Gal, 2017). Uncertainty that originates from noise in observations, meaning for example missing measurements, irrelevant data or mislabeled examples, is called *aleatoric* uncertainty. The other type of uncertainty is called *epistemic* uncertainty. It refers to uncertainty that results from the model. In particular in image classification, approaches such as Bayesian deep learning can be applied and extended to handle and explicate uncertainties.

For enabling such conceptual properties, an integration of concepts, approaches, techniques and measures from a variety of disciplines is necessary as depicted in Figure 4. We refer to and extend a proposal from the Defense Advanced Research Projects Agency (DARPA) to elaborate cAI emphasis by showing relevant research disciplines and its relationships to AI (Gunning, 2016). In this context, the emphasis of cAI is defined as an overlapping of the disciplines AI, Human Computer Interaction (HCI) and End User Explanation with its interdisciplinary techniques and approaches like visual analytics, interactive ML and dialog systems. Furthermore, domain requirements, legal as well as ethic aspects participate and contribute to an overall understanding of cAI.

## 3. Fundamentals of cAI Transition Applied to Medicine

The relevance of cAI becomes clear when ML is applied to medicine. In common, medical sub-disciplines rely on high sensitivity and specificity of diagnostic decisions. In order to choose the right therapy and to avoid delays in treatment caused by initial misdiagnosis, neither false alarms nor miss outs are desirable. Several recent studies show that ML can help to increase the accuracy of diagnosis (Weng et al., 2017; Haenssle et al., 2018; Hu et al., 2019). Applying ML therefore has the potential to save lives and resources. Especially sub-disciplines that are based on image processing and classification, like histology, could benefit from high performing approaches such as convolutional neural networks (Buetti-Dinh et al., 2019). However, since these approaches remain a black-box, medical experts cannot comprehend why a certain classification was performed and thus convolutional neural networks should not be applied in decision-critical tasks unless their predictions are made comprehensible and robust. Even though an ML approach shows a high classification accuracy, it still might be biased (Gianfrancesco et al., 2018). In the following sections we present the cornerstones as well as some specific approaches for improving comprehensibility of expert companions for the medical domain.

### 3.1. Explanation Generation and Visual Analytics

Visual analytics techniques, which in our transition framework from Figure 4 are located at the intersection of AI and End User Explanation, can be used to provide visualizations that are helpful for humans to interpret according models or its results. Therefore, human comprehensible End User Explanations need to be built on top of formal explanations by considering and using knowledge from psychological and philosophical investigations. These, inter alia, strive for the generation of explanations understandable for humans and for an efficient communication by conveying the causal history of the events to be explained (Lewis, 1986). As a consequence, most state of the art explanation generators try to use visualization techniques in order to generate explanations that are relevant both to the implicit questions of the explainees as well as to their mental models (Miller, 2019). A prominent xAI technique, which allows for local, model-agnostic and *post-hoc* interpretations by approximating black-box models locally in the neighborhood of predictions of interest, was proposed by Ribeiro et al. (2016). LIME uses a local linear explanation model and can thus be characterized as an additive feature attribution method (Lundberg and Lee, 2017). Given the original representation *x* ϵ ℝ^*d*^ of an instance to be explained, *x*′ ϵ {0, 1}^*d*′^ denotes a binary vector for its interpretable input representation. Furthermore, let an explanation be represented as a model *g* ϵ *G*, where *G* is a class of potentially interpretable models like linear models or decision trees. Additionally, let Ω(*g*) be a measure of complexity of the explanation *g* ϵ *G*, for example the number of non-zero weights of a linear model. The original model that we are searching explanations for is denoted as *f* : ℝ^*d*^ → ℝ. A measure π_*x*_(*z*) defining the locality around x is used that captures proximity between an instance *z* to *x*. The final objective of LIME is to minimize a measure $L\left(f,g,\pi_{x}\left(z\right)\right)$ that evaluates how unfaithful *g* is in approximating *f* in the locality defined by π_*x*_(*z*). Striving for both interpretability and local fidelity, a LIME explanation is obtained by minimizing $L\left(f,g,\pi_{x}\left(z\right)\right)$ as well as keeping Ω(*g*) low enough to be an interpretable model:

(1) $$\begin{array}{r} \xi \left(x\right)=\underset{g\epsilon G}{\arg\min}\;L\left(f,g,\pi_{x}\left(z\right)\right)+\Omega \left(g\right) \end{array}$$

**Figure 5:**
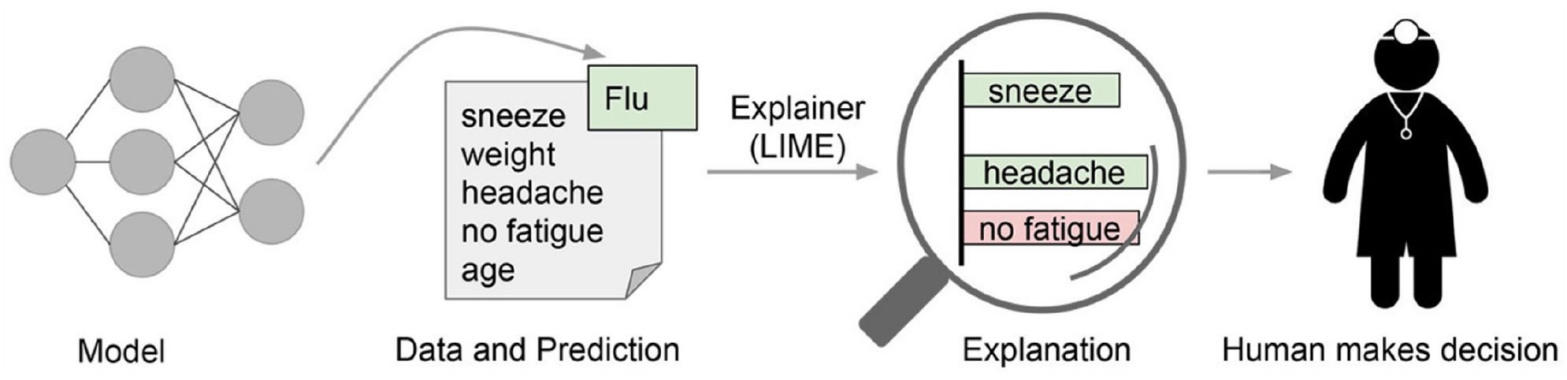
LIME: Explaining individual predictions in the medical context (Ribeiro et al., 2016).

For being a model-agnostic explainer, the local behavior of *f* must be learned without making any assumptions about *f*. This is achieved by approximating $L\left(f,g,\pi_{x}\left(z\right)\right)$, drawing random samples weighted by π_*x*_(*z*). Having drawn non-zero elements of *x*′ uniformly at random, a perturbed sample *z*′ ϵ {0, 1}^*d*′^ is obtained. Recovering *z* from *z*′ and applying *f*(*z*) then yields a label, which is used as label for the explanation model. The last step consists of optimizing Equation (1), making use of dataset Z that includes all perturbed samples with the associated labels. Figure 5 depicts an exemplary explanation process of LIME in the medical domain that explains why a patient was classified as having the flu by portraying the features *sneeze* and *headache* as positive contributions to having the flu, while *no fatigue* was considered as evidence against the flu. Other techniques for generating explanations, especially for concrete predictions of neural networks, comprise Layer-wise Relevance Propagation (LRP), which identifies properties pivotal for a certain prediction, as well as neural network rule extraction techniques like Neurorule, Trepan, and Nefclass (Beasens et al., 2003; Lapuschkin, 2019). All of these approaches share in common that they either provide explanations in terms of visualizations by showing the most important features relevant for a single prediction or by providing rules that are represented as decision table. As an example, Binder et al. (2018) developed an approach for predictive learning of morphological and molecular tumor profiles. In addition to purely focusing on prediction accuracy, the authors applied LRP in order to analyze the non-linear properties of the learning machine by mapping the results of a prediction onto a heatmap that reveals the morphological particularities of the studied pathological properties. Hägele et al. (2019) analyzed histopathological images and applied LRP for visual and quantitative verification of features used for prediction as well as for detection of various latent but crucial biases using heatmapping.

Out of such explanations and visualizations, experts might get valuable interpretations, but to even improve interpretability especially for lay humans it could be helpful to include other explanation modalities. As an example, combining visual explanations with natural language explanations as well as allowing for more interactivity between ML systems and users could further improve trust in the system. Additionally, in our opinion the process of transferring and presenting generated explanations should be made up in a way such that semantic level of detail as well as semantic context are aligned between the ML system, the explanation system and the human user. Therefore, our transition framework includes an interdisciplinary, psychologically motivated research area that deals with *End User Explanations*. Psychological insights into the process of generating and communicating explanations can be derived from explanatory understanding (Keil, 2011). According to that, explanations reveal a transactional nature and communicate an understanding between individuals. Additionally, as humans adapt stances or modes of construal (Dennett, 1987) that frame explanations, the latter ones reveal an interpretative nature and require humans to perform mental calculations in order to understand explanations. Therefore, the authors from Sloman et al. (1998) and Ahn et al. (2000) name circularity, relevance and especially coherence as further important dimensions that guide systematic evaluation of explanations. Coherence in the domain of explanations describes the fact that humans prefer explanatory features within induction, which are most causally interdependent on others and therefore coherent. Furthermore, explanations are deemed relevant and informative when being presented to humans at the correct level of semantic detail. In essence, high quality explanations stick together and represent an internally consistent package, whose elements form an interconnected, mutually supporting relational structure (Gentner and Toupin, 1986; Thagard, 2000).

Many state of the art explanation systems, especially those based on perturbations, reveal some significant drawbacks. One of them is the fact that they sample instances around the instance to be explained by drawing samples uniformly at random. Doing so they ignore feature dependence when sampling from a marginal distribution (Molnar, 2019). Thus, there is a high chance that subsequent explanation strategies put too much weight on unlikely data points and are therefore susceptible for extrapolation. In such a case, explanations can then easily be misinterpreted. As a further consequence, context between the explanation features is not considered, yielding explanations, where humans have to perform many mental calculation steps in order to interpret and understand the explanations properly. Another potential problem is described by the authors (Alvarez-Melis and Jaakkola, 2018), namely potential instabilities of explanations manifesting in great variances for explanations of two close data points. Due to the random-sampling-step, one of the necessary concepts from our transition framework, namely confidence, is often violated. The authors from Arrieta et al. (2020) refer to confidence as a generalization of robustness and stability, which are themselves also motivated by the problem of missing reproducibility of the ML predictions as well as the according explanations. Finally, missing context between explanation features can lead to a lack of semantic interactivity between ML system and human users, since humans think and explain via *semantic coherent concepts* that the explanation systems are often not able to deal with.

**Figure 6:**
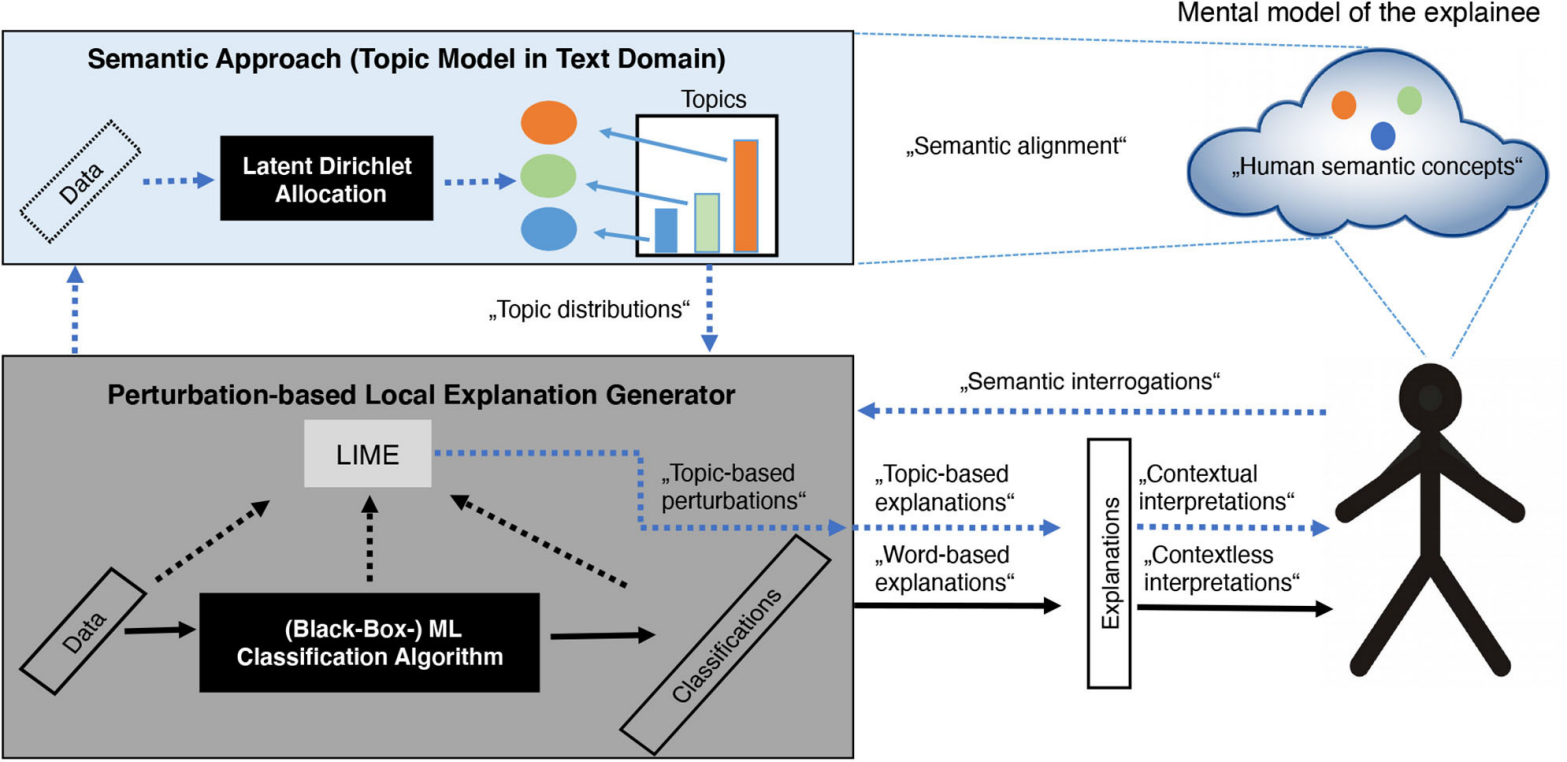
Integration of a ML classification algorithm, an explanation system like Local Interpretable Model-agnostic Explanations (LIME) as well as a *semantic* approach: Black arrows represent the classical way of generating and communicating explanations in a model-agnostic and perturbation-based way, while the blue dotted arrows show the explanation process integrating a semantic approach.

As LIME is a representative of perturbation-based explanation systems and constitutes state of the art within xAI for image as well as for text classification (both of which are highly relevant within the medical domain), we propose an architecture to overcome some of the drawbacks mentioned above especially for text classification combined with LIME. Therefore, we propose the integration of (a) a ML classification algorithm, (b) an explanation system like LIME as well as (c) a *semantic* approach. In text domain, the latter is represented as a text modeling approach, in specific a topic modeling approach like Latent Dirichlet Allocation (LDA) that captures semantic and contextual information of the input domain. The goal of this integrated architecture (as illustrated in Figure 6) is to provide the basis for *coherent* and therefore *human-interpretable, contextual* explanations and to enable insights into the classifier's behavior from conceptual point of view. Harnessing semantic and contextual meta-information of the input domain by learning human-interpretable latent topics with LDA enables a Perturbation-based Local Explanation Generator like LIME to sample from a realistic local distribution via topic-based perturbations. As a result, topic-encoded explanations are obtained, which allow humans to recognize correlations (context) and to perform interpretations more intuitively by aligning the encoded semantic concepts with their mental model. Another interesting property of a combination of an explanation system combined with a semantic approach is its *semantic interrogation ability*. When it comes to the point, whether to trust a ML classifier, potential questions to be answered could be: “Does a classifier behave in a manner that is expected by humans?” or “How much does a classifier resemble human intuition”. A semantically enriched architecture enables humans to generate documents that reveal a specific semantic content (represented as a mixture of certain topics and according words) as well as semantic structure. Presenting those user-specified documents to the classifier and receiving the according classifications, human users can interact with the classifier through an explanation system via *semantic interrogations*. These will be answered by the classification system with topic-based explanations allowing the user to interpret them in terms of human semantic concepts.

Applying such an architecture to the medical domain can help with improving and explaining automatic recognition of medical concepts in (un-)structured text (i.e., patient records), which is a complicated task due to the broad use of synonyms and non-standard terms in medical documents (Arbabi et al., 2019). In essence, better reproducibility of explanations can be achieved by reducing randomness during perturbation by the integration of *semantic sampling* that also allows to generate contextual explanations, which in turn can be interpreted by humans more intuitively.

### 3.2. Verbal Explanations

As described in the previous subsection, providing visual explanations and semantics helps to increase the interpretability of opaque classifiers. In addition, natural language explanations constitute an important explanation modality, since, for their expressiveness, they capture complex relationships better than visualizations (Finzel et al., 2019; Rabold et al., 2019; Schmid and Finzel, 2020) and increase comprehensibility (Muggleton et al., 2018).

**Figure 7:**
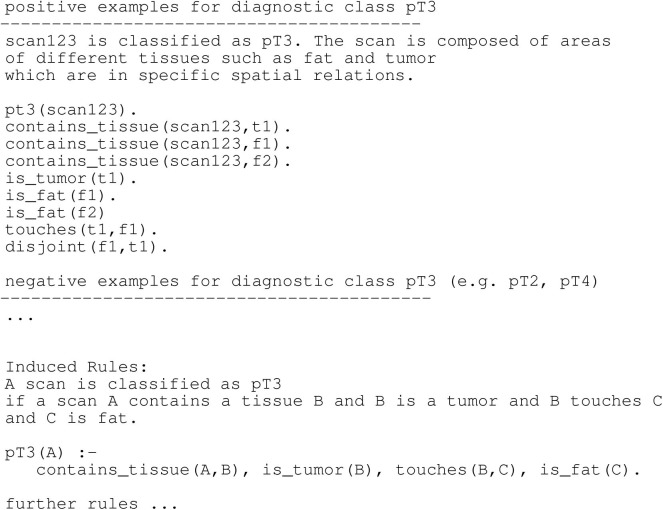
Training examples and learned rules for a hypothetical diagnostic domain of colon cancer (Schmid and Finzel, 2020).

In our transition framework (see Figure 4) we include verbal explanations at the intersection of End User Explanation and HCI. We show in the following paragraphs that natural language plays a key role in enhancing the comprehensibility of classifier results and that it is an important modality to allow for meaningful interactions between the classifier and a medical expert. Medical diagnosis often relies on the visual inspection of image- or video-based data, such as microscopy images, cardiograms or behavioral data from videos (Schmid and Finzel, 2020). In many cases, diagnostic decisions are not made solely based on the mere occurrence or absence of symptoms and abnormalities. The analysis of images and videos often takes into account spatial information and spatial relationships between the entities of interest. Visual explanations are limited with respect to representing relations. Visualizations, such as heatmaps and superpixel-based highlights are restricted to presenting conjunctions of information, i.e., (co-)occurrence of entities of interest. Although negation can be encoded with the help of the color space (e.g., in LRP-based heatmaps, where highlights in a color opposite to positive relevance indicate that some important property is missing), interpreting and semantically embedding which property is negated in comparison to the properties of contrasting classes, remains the task of the human expert. Therefore, enhancing understanding by visual explanations is limited, since the latter can only be interpreted with respect to positions of entities and given conjunctions of highlights encoded by the color space. They lack to express more complex relationships, such as spatial relations between two or more entities. Arbitrary relationships and special cases of relational concepts, for example recursion, can be better represented in natural language. Therefore, verbal explanations better qualify for giving insights into causal chains behind classification and thus diagnostic problems. This is especially important, since expert knowledge is often implicit and making it explicit can be hard or even impossible for experts. Particularly interesting are therefore systems that are capable of learning relational rules, which can then be translated into natural language expressions for generating verbal explanations. As presented for example in Schmid and Finzel (2020), spatial relationships are considered in the analysis of microscopy images to verbally explain the classification of the depth of invasion for colon tumors. In this use case, not only the occurrence of tissues, but also the complex spatial relationships between different types of tissue must be taken into account. For example, if tumor tissue has grown passed muscle tissue and already invades fat, the tumor class is more critical compared to a tumor that resides within tissue of the mucosa (Wittekind, 2016). As further pointed out in Schmid and Finzel (2020), ML approaches should therefore be able to reveal which relationships lead to a certain classification. Furthermore, relationships should be communicated in a comprehensible way to medical experts and this can be achieved with the help of natural language explanations. In their project the authors utilize Inductive logic programming (ILP) to implement a comprehensible explanation interface for a *Transparent Medical Expert Companion*, a system that explains classification outcomes of black-box and white-box classifiers and allows for interaction with the medical expert. ILP is an ML approach that produces output that can be transformed into verbal explanations for classification outcomes. In the *Transparent Medical Expert Companion*, microscopy scans are classified either by human experts or by an end-to-end black-box ML system. In the given example (see Figure 7), target class is tumor class *pT3*. Scans that are classified as *pT3* are positive examples, scans with different classification are negative. Learning can be realized by a one-against-all-strategy or separated in different sub-problems, such as discriminating one target class from the most similar alternative classes. An ILP system can now be used to learn over the given examples. In Figure 7, an illustration for one learned rule is given. A new scan is classified as *pT3* if it fulfills all components of the rule. In order to transform such rules into verbal explanations, methods similar to those introduced in the context of expert systems can be utilized (Schmid and Finzel, 2020).

**Figure 8:**
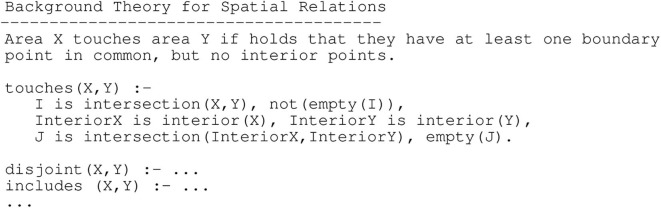
Background theory with domain rules for a hypothetical diagnostic domain of colon cancer (Schmid and Finzel, 2020).

In addition, experts can still provide their knowledge to the algorithm, as illustrated in Figure 8, where an exemplary spatial relationship *touches* is defined in the background knowledge and can thus be found by the algorithm in the data if relevant to the classification of *pT3*. It has been shown that due to the implicitness of expert knowledge and variants in how health symptoms manifest, it is easier for an expert to determine why a certain example belongs to a diagnostic class rather than describing the class in its entirety (Možina, 2018). Rules learned by ILP can be traced, meaning that they can be applied to the background knowledge, which contains the data from examples like a data base. This way, the learned program, consisting of the learned rules and the data base, can explain its reasoning to the human expert. This is done by showing the output from the chain of reasoning steps, as it has been implemented for example in the diagnostic system MYCIN (Clancey, 1983). Traces can be translated into natural language expressions and then used in explanatory interaction in the form of a dialog between the system and the human expert, where the expert can ask for clarification in a step-wise manner. Research on how these dialogs could be implemented are concerned with rule-based argumentation (Možina et al., 2007), argumentation schemes and the form of argumentative input [e.g., free-form, structured, or survey-based (Krening et al., 2017)]. Finally, natural language is the basis for more expressive correction of classification decisions, which will be discussed in the next subsection.

### 3.3. Interactive Machine Learning

Since medical knowledge changes steadily (which can lead to a bias in models learned on outdated data), ML approaches are needed that are able to adapt or that can be adapted easily by medical experts. This is where interactive ML comes into play. The main motivation of *human-in-the-loop*-based interactive ML is to build systems that improve their learning outcome with the help of a human expert who interacts with them (Holzinger, 2016). The human expert interacts for example with the data to improve the prediction outcomes and helps to reduce the search space through her expertise. The vision behind interactive ML is to “enable what neither a human nor a computer could do on their own” (Holzinger, 2016). Still, in the context of comprehensible interfaces for machine learned classifiers, mostly explanations are unidirectional—from the AI system to the human (Adadi and Berrada, 2018). Therefore, there exists a big potential for the medical domain to improve diagnosis with the help of developing new interactive ML approaches. State of the art approaches include systems where the human expert labels an example that was chosen by the algorithm according to some preference mechanism. In adherence to the so-called *active learning* paradigm, the system learns from the interaction with the user and may produce better prediction outcomes afterwards. Likewise experts can change labels of incorrectly classified examples or may add new examples with new labels in an incremental way. Furthermore, there exist approaches where the user has the possibility to indicate which features are relevant or irrelevant to a certain classification. An exemplary system is the EluciDebug prototype (Kulesza et al., 2015) for categorizing emails. After putting an incoming email into a certain folder, the system lists the words that have been considered as relevant. The user can adjust weights, for example to decrease the importance of words in order to remove them from decision rules. With the help of active learning particularly the data bias can be controlled by the human expert. There exist approaches and proposals for systems that offer explainable classification and allow user feedback in form of corrections beyond re-labeling and feature weighting that in a next step are used to adapt the machine learned model. One of the first approaches is the interactive learning system Crayon that enables the user to correct a classification of objects in an image by simply re-coloring some of the misclassified pixels (Fails and Olsen, 2003) to retrain the model. A second approach is named CAIPI (Teso and Kersting, 2019). It combines querying an example image, making a local prediction with a black-box learner and explaining the classification with an xAI approach, allowing the user to give feedback in the form of pixel re-coloring and re-labeling of false positives. Although both approaches offer promising ways of user interaction, they only take into account pixel-based visual information, omitting textual or relational information that might be relevant for expert decision making.

Interaction can be taken a step further. In domains where class decisions are based on complex relationships, interaction that allows for correction of relational models can improve the human-AI partnership (Schmid and Finzel, 2020). It has been shown also in other domains of AI that explanations can be used to revise current models (Falappa et al., 2002). A bi-directional exchange between an ILP system and a human expert is realized in the exemplary system *LearnWithME* (Schmid and Finzel, 2020) that integrates the principle of ME.

The aim behind the application *LearnWithME* is to provide medical experts a companion system for improved diagnosis. Companion systems serve as assistants to support humans in their daily or work routine. Adaptive machine learning, which incorporates interaction with the human and incremental learning, is suitable to enhance such companions (Siebers and Schmid, 2019). Furthermore, cognitive conditions imposed by the context of use and the user should be considered (Cawsey, 1991, 1993).

**Figure 9:**
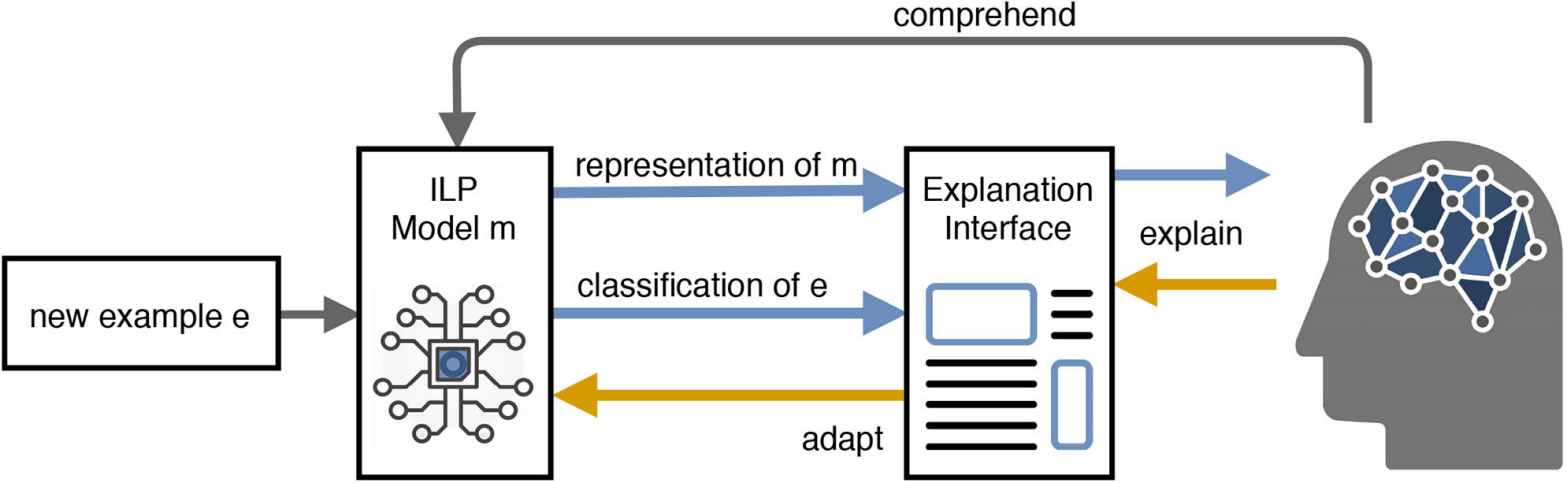
Model of a mutual explanations system (Schmid and Finzel, 2020).

Accordingly, the concept of *Mutual Explanations* is a cooperative, interactive and incremental act of information exchange between humans and machines with the goal to improve the joint performance of the involved partners in classification problems. The process of explanation refers (1) to providing arguments that make simple and complex relations, which apply to the domain of interest, explicit and (2) to integrating corrective explanations into existing internal models in order to adapt these (Schmid and Finzel, 2020). A model of such a ME system, which allows for bidirectional communication via explanations as well as interactive ML (corrections for model adaptation), is given in Figure 9: Starting with an initial ILP model, a new instance *e* is classified. The class decision for *e* is presented to the human who can accept the label or ask for an explanation. The explanation can be accepted or corrected with the help of defining constraints over the verbalized model at class level or at the level of the instance explanation.

Learning expressive, explicit rules rather than a black-box classifier has the advantage that generation of verbal explanations is quite straight-forward. However, in image-based medical diagnosis, it is clearly desirable to indicate a system's decision directly in the image. Often, only a combination of visual highlighting and verbal relational explanations allows to convey all information relevant to evaluate a decision. We believe that our cAI framework therefore provides a guideline to the development of interpretable systems for the medical domain by integrating visual and verbal explanations as well as interactive machine learning at the level of model adaptation through corrective feedback.

## 4. Conclusion

In the course of this paper we described why comprehensibility and interactivity will be crucial properties of modern ML systems in many application domains and especially for the task of designing transparent expert companions for the medical domain. Since thoughts on improved interpretability started to get considerable attention and many related concepts and terms have not been clearly defined yet, we introduced the term and concept of *Comprehensible Artificial Intelligence*. By describing and putting the basic cognitive concepts for cAI research and practice in relation, we were able to assign and discuss many current related research questions in an integrated manner from conceptual point of view. Furthermore, we gave a brief summary of connected interdisciplinary research areas and their overlappings, jointly being able to address many of the shortcomings mentioned in current literature. An integrated cAI transition framework was introduced revealing the guiding principles for exploring and implementing ML approaches that humans have trust in and can interact with. Our framework can be considered by developers and practitioners as a guideline to identify necessary concepts and possible solutions for their individual medical context. To the best of our knowledge, this has not been done yet beyond the scope of a literature review. We based our transition framework on theoretical foundation, derived practical implications and gave examples for possible solutions.

Following along our framework during some prototypical use cases, we identified *Semantic Alignment* between ML classifiers and human users, which is often overlooked in current approaches, as necessary prerequisites for comprehensibility as well as interactivity. Considering psychological insights from explanatory understanding, we proposed to properly account for the individual mental models of the explainees by integrating a semantic approach into a classification pipeline and presenting explanations at an appropriate level of semantic details. Especially when using black-box-algorithms and perturbation-based explanation systems, such an architecture can be used to enable realistic perturbations that reflect the underlying joint distribution of the input features and to generate meaningful, useful and more reproducible explanations. Our claim is that semantic and contextual information provided by the input domain must be taken into account during explanation generation and presentation, such that coherent and human-interpretable explanations are obtained bringing to light logical as well as causal correlations. For the task of classifying and explaining text documents being made of medical concepts, we describe a process that allows to find local topic-based explanations using topic models like Latent Dirichlet Allocation together with LIME. To even increase comprehensibility of explanations in terms of expressiveness, we suggest to include other explanation modalities as well. In addition to visual inspection as often conducted in medical diagnosis, verbal explanations and according methods to directly obtain them from classification systems are analyzed and shown exemplary with the help of Inductive Logic Programming. Furthermore, we provide the prospect of *Semantic Interrogations* to compare a classifier's semantic classification ability with human semantic concepts. As a kind of overall realization concept this paper introduces ME that in our opinion can provide a valuable basis for providing bidirectional information exchange between humans and machines. Summarizing and integrating all mentioned concepts in a single framework shall guide practitioners when attempting to create interactive, transparent and comprehensible ML systems that even laymen can interpret and build trust in.

Although many topics have been discussed with regard to the medical domain, the main points remain valid across different application domains. Adapting these approaches to the context of the individual problem as well as assessing explanations' quality quantitatively as well as qualitatively in a pragmatic way, these are the points that in our opinion constitute main future demands on cAI. Trying to anticipate ML's future in research and practice, we request for a stronger interdisciplinary thinking on cAI. This implies not just researching for formal explanations for ML systems and decisions, but trying to allow for an efficient generation and transportation of interpretation artifacts to human users considering disciplines like explanatory understanding. It shall allow humans to gain a deeper understanding leading to improved interpretations forming the basis for transparent and comprehensible AI that we refer to as cAI.

## Author Contributions

SB, BF, and US made substantial contributions to conception and design of their approach. All authors involved in drafting the manuscript or revising it critically for important intellectual content, gave final approval of the version to be published, read, and approved the final manuscript.

## Conflict of Interest

The authors declare that the research was conducted in the absence of any commercial or financial relationships that could be construed as a potential conflict of interest.
